# The Effects of Rhythmicity and Amplitude on Transfer of Motor Learning

**DOI:** 10.1371/journal.pone.0046983

**Published:** 2012-10-03

**Authors:** Mor Ben-Tov, Shelly Levy-Tzedek, Amir Karniel

**Affiliations:** 1 Department of Biomedical Engineering, Ben-Gurion University of the Negev, Beer-Sheva, Israel; 2 The Edmond and Lily Safra Center for Brain Sciences (ELSC), The Hebrew University, Jerusalem, Israel; 3 Department of Biomedical Engineering, Ben-Gurion University of the Negev, Beer-Sheva, Israel; Harvard University, United States of America

## Abstract

We perform rhythmic and discrete arm movements on a daily basis, yet the motor control literature is not conclusive regarding the mechanisms controlling these movements; does a single mechanism generate both movement types, or are they controlled by separate mechanisms? A recent study reported partial asymmetric transfer of learning from discrete movements to rhythmic movements. Other studies have shown transfer of learning between large-amplitude to small-amplitude movements. The goal of this study is to explore which aspect is important for learning to be transferred from one type of movement to another: rhythmicity, amplitude or both. We propose two hypotheses: (1) Rhythmic and discrete movements are generated by different mechanisms; therefore we expect to see a partial or no transfer of learning between the two types of movements; (2) Within each movement type (rhythmic/discrete), there will be asymmetric transition of learning from larger movements to smaller ones. We used a learning-transfer paradigm, in which 70 participants performed flexion/extension movements with their forearm, and switched between types of movement, which differed in amplitude and/or rhythmicity. We found partial transfer of learning between discrete and rhythmic movements, and an asymmetric transfer of learning from larger movements to smaller movements (within the same type of movement). Our findings suggest that there are two different mechanisms underlying the generation of rhythmic and discrete arm movements, and that practicing on larger movements helps perform smaller movements; the latter finding might have implications for rehabilitation.

## Introduction

The extent of the overlap in the control mechanisms of rhythmic and discrete movements has been explored in several studies, both on the behavioral [1], [2], [3], [4] and on the neural level [5]. Hogan and Sternad [6] defined precise mathematical kinematic measures for discrete and rhythmic movements. Using these definitions, we have previously shown [7], [8] that in a spatiotemporally defined task, the frequency of the movement can determine the type of movement one performs. High-frequency movements are highly rhythmic and low-frequency movements are more discrete-like. Here, we used the same experimental paradigm to test whether there exists transfer of learning between the two movement types. In addition, we explored the effect of movement amplitude on the transfer of learning.

Previous studies have shown effective learning transfer between different types of movement. Abeele and Bock [9] found a considerable transfer of adaptation between pointing and tracking tasks. In a prism adaptation task, Kitazawa et al. [10] found the almost full transfer from fast to slow reaching movements but partial transfer from slow to fast movements. Dean et al. [11] showed that in a sequences-learning task, practice on a large-scale movement leads to a more effective transfer to a smaller-scale movement than vice versa. In addition, in a visuomotor rotation task a transfer of learning was found only from discrete to rhythmic movement, but not the other way around [2] These findings indicate that (1) there can be a transfer of learning between different movement categories (2) this transfer is not necessarily bi-directional.

Two recent studies explored the relationship between rhythmic and discrete movements. Howard et al. [1] associated each type of movement to a different force perturbation and showed that the subjects could learn both force fields without interference. Ikegami et al. [2] used a learning transfer paradigm with a visuomotor rotation task and showed a partial transfer of learning from discrete to rhythmic movement but not vice versa; they also showed that longer dwell times between one discrete movement to the other minimize the amount of this transition. Therefore we hypothesize that perhaps discrete and rhythmic movements are being controlled by at least partially separated mechanisms.

The goal of this work is to examine which aspects of the movement (amplitude, rhythmicity or both) are important for learning to be transferred from one type of movement to another. We propose two hypotheses which we test in the following set of experiments, in which subjects performed repetitive arm movements with different amplitudes and frequencies: (1) rhythmic and discrete movements are controlled by (at least partially) separate mechanisms; therefore we expect to see no transfer of learning between the two types of movements. If we do find some transfer of learning, it will be from discrete point-to-point movements to rhythmic movement (see Ikeagami et al., [2]); (2) within each movement type (rhythmic/discrete), there will be asymmetric transfer of learning from larger movements to smaller ones [11].

## Methods

### Participants

60 young healthy adult right-handed participants (Age: 24.8±2.1 years; 29 females, 31 males) and 10 healthy right-handed children (Age 9.4±1.1, 9 females, 3 males) were tested using their right arm. Adult Participants were randomly assigned to one of ten experimental groups, and children were randomly assigned to one of two experimental groups. All participants gave their informed consent to participate, after signing the informed consent form, and one of the parents of each child also signed the appropriate form as stipulated by the Institutional Helsinki Committee, Beer-Sheva, Israel.

### Experimental Apparatus

**Figure 1:**
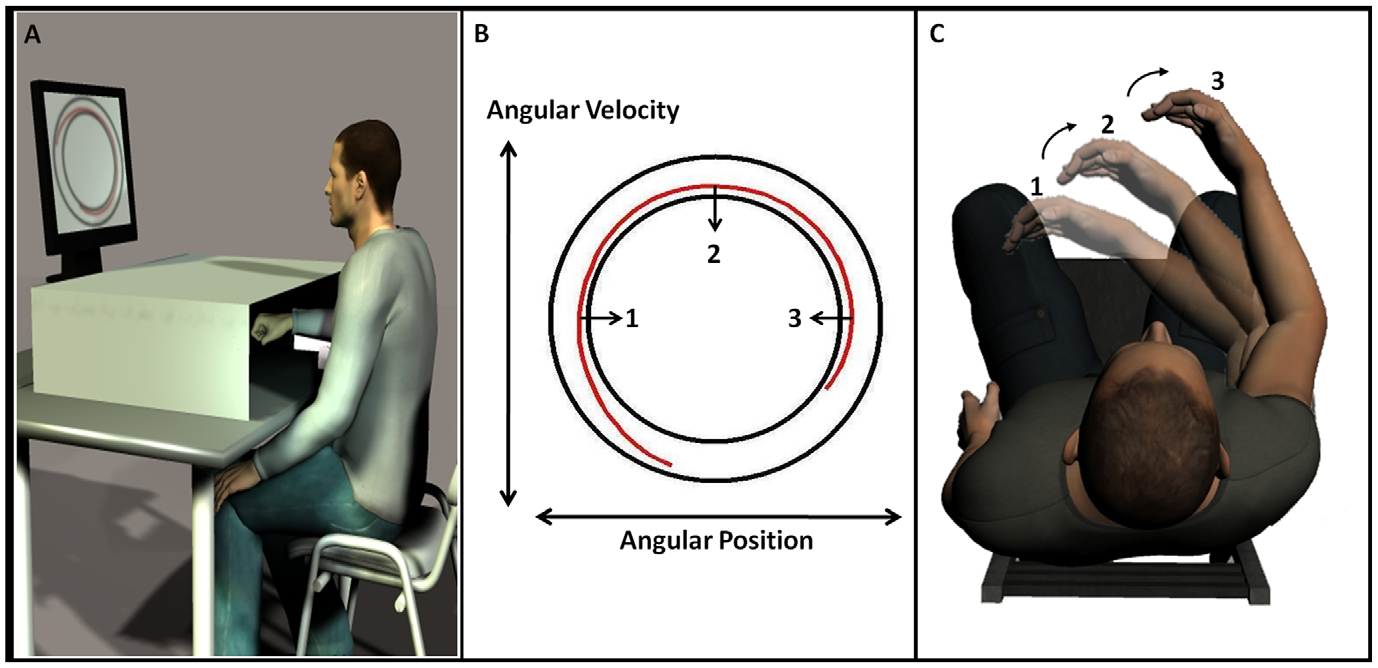
Experimental setup. (A) The experimental apparatus illustrated. The participant’s forearm is placed on a forearm support, allowing one-dimensional horizontal movements. The forearm is occluded from view, and visual feedback of the movement is given on the screen, in the form of a phase-plane display: the black ellipses represent the required limits of the movement's amplitude and speed and the red line represents the trace of the participant's movement on the phase plane (B) The phase-plane display, showing angular velocity vs. angular. The area between the two black ellipses is the region within which participants are required to maintain the trace of their movement. The red trace corresponds to the participant's movement in the phase plane. The numbers “1” and “3” represent the maximum flexion and extension, respectively, the number “2” represents the location where the movement speed peaks (C) A top view of the participant's forearm at three points along the movement trajectory (the numbers 1–3 correspond to the phase-plane locations marked in panel B.

The participants sat on a straight-back chair and placed their right arm on a forearm support, consisting of a wrist brace strapped to an arm rest. The forearm support was connected to the shaft of a rotary incremental encoder with a position resolution of 0.002 degrees per count. Data were recorded at 200 Hz. A computer screen was used to display the position and the velocity of the forearm. A large, opaque plastic cover was placed parallel to the table, and above the apparatus, such that during the experiment, the participant’s forearm was occluded from view (see Figure 1A).

### Experimental Protocol

The participants were instructed to perform horizontal flexion/extension movements with their forearm. They were presented with a phase-plane display of their forearm motion; the horizontal axis represented the angular position and the vertical axis represented the angular velocity (see Figure 1B). The task was to move the forearm such that the trace of the movement on the phase plane would remain within a dictated region: a doughnut shape formed from two ellipses displayed on the screen (see Figure 1B [7], [8], [12], [13], [14]). Each ellipse corresponds to a sinusoidal motion about the elbow, with the nonzero width of the doughnut shape allowing for a range of amplitudes and speeds, and therefore frequencies [13]. Participants could see the doughnut-shaped target region, as well as a trace corresponding to their own forearm motion (see Figure 1B). At the beginning of each experiment, participants performed four trials of flexion/extension movements without any restrictions or visual feedback on their movement, in order to become familiar with the experimental system.

We conducted a total of ten experiments. Experiments 1–8 were aimed at testing the effect of rhythmicity and amplitude on the transfer of motor learning and experiments 9–10 served as controls, to make sure that speed does not affect the results when transitioning between the two movement types. Each experiment consisted of 3 blocks, with 10 trials in each block for total of 30 trials per experiment. In experiments 1–8 each trial was 15 seconds long. At the beginning of each trial, the initial position of the participant's forearm was measured and the experiment's coordinate system was calibrated according to this position, e.g. no matter what was the exact initial position of the forearm, each subject began the movement with position equals to zero. In each trial participants had to perform back and forth flexion-extension movements of their forearm until the computer screen changed it color and the trial was over. There were short breaks between one trial to the other (a few seconds). If a participant felt tired, he or she could rest as long as they wished between two consecutive trials. The majority of the participants did not ask for breaks during the experiment. There was no break between blocks. The blocks differed from one another by the required amplitude and/or frequency of the movement. The eight block sequences corresponding to eight experimental groups 1–8 were:

1. DS – DS – RS
2. DB – DB - RB
3. RS – RS – DS
4. RB – RB – DB
5. DS – DB – RS
6. DB – DS - RB
7. RS – RB – DS
8. RB – RS - DB

Where D stands for discrete movement (central frequency = 0.35 Hz), R stands for rhythmic movement (central frequency = 1.7 Hz), S stands for small movement (central amplitude = 20 degrees) and B stands for big movement (central amplitude = 40 degrees). Discrete and rhythmic frequency values were determined according to the results reported in [7], [8]. In the discrete conditions participants perform an average of 5 cycles per trial and in the rhythmic conditions 25 cycles. Changes in the amplitude and frequency of the movement resulted in different movement speed. The central values of the peak speed were: 22, 44, 107 and 214 degrees per second for blocks DS, DB, RS and RB respectively. We determined the doughnut shape as follows: after determining the central amplitude and frequency, we calculated the desired peak speed, which affects the vertical axis of the ellipse. Then we set the inner ellipse to match the values of 15% less than the central amplitude and speed and the outer ellipse to 15% more of these values. In these experiments the display on the screen was the same throughout the experiment and the participants could not predict when the movement requirements were about to change during the experiment.

Experiments 1–4 explore the effect of rhythmicity alone on the transfer of learning. In these experiments the first two blocks were identical in terms of rhythmicity and amplitude, and only one transition occurred between the second and third experimental blocks. In that transition only the type of movement changed (for example, in experiment 1, the first two blocks require small discrete (DS) movements and the third block requires small rhythmic ones (RS)). Based on our assumption that there are two different mechanisms for the control of rhythmic and discrete movements, we expect there will be no transfer of learning between these two types of movement.

Experiments 5–8 contained two transitions; the first (the transition between the first and second experimental blocks) was designed to explore the effect of changing the amplitude within the same type of movement (rhythmic/discrete). For example, in experiment number 5 participants performed small discrete (DS) movements and then big discrete (DB) movements. In the second transition (the transition between the second and third experimental blocks), both amplitude and type of movement (rhythmic/discrete) were changed (e.g. the transition between big discrete (DB) movements to small rhythmic (RS) movements in experiment 5). This second transition between blocks, where both amplitude and rhythmicity were changed, was designed to explore whether the change in amplitude together with a change in the rhythmicity of the movement affect the transfer of learning.

In the two control experiments, we sought to equate both amplitude and speed across all blocks in each experiment, with the sole modification being the rhythmicity of the movement, as defined by the presence or absence of distinct pauses (see Hogan & Sternad [6]). Note that in experiments 1–8, a change in rhythmicity was accompanied by a change in movement speed, and we wanted to verify that if there is no transfer of learning between the blocks in those experiments, it is not the result of the change in speed between them. The block sequences corresponding to experiments 9–10 (control experiments) were:

1. TDB - TDB - RB
2. RB - RB - TDB

**Figure 2:**
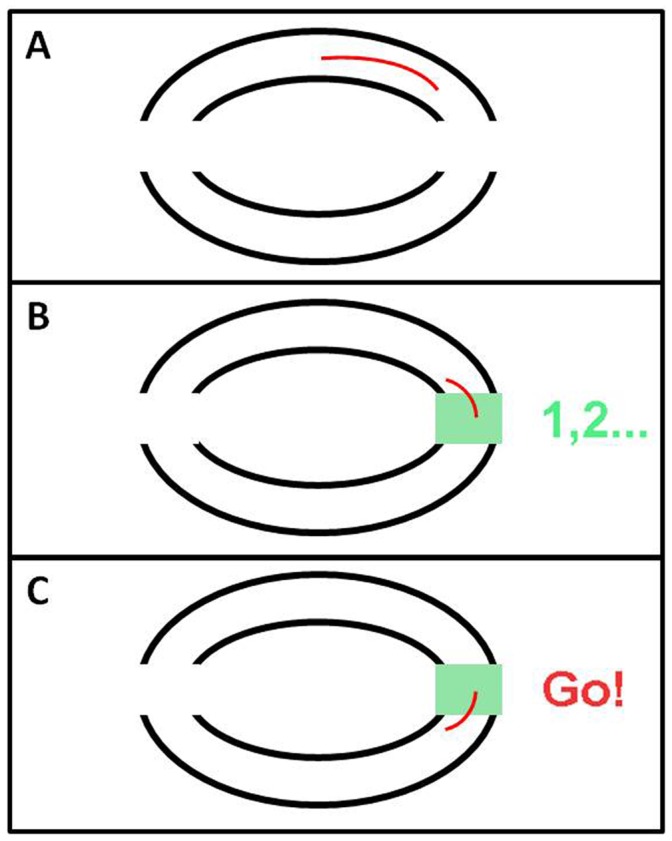
The display on the screen in TDB trials. (A) Two white squares at the two movement extremes (B) When the participant's movement trace reached an end-point, the corresponding square become green. (C) After a 2-sec pause, the word “Go!” appeared, and the participant was required to make another discrete movement.

In these experiments the rhythmic condition was the same as described above and the “truly discrete” (TD) condition was a point to point movement, with the same required peak speed and amplitude as in the RB block, but with distinct pauses between movements (where each movement consisted of either flexion or extension of the forearm). In the TD blocks, two white squares were displayed on the left and on the right sides of the elliptic doughnut (see Figure 2A). When the participant’s movement trace reached one of the squares, the square changed its color to green (see Figure 2B), and the participant had to wait for 2 seconds until the word “Go!” appeared next to that square (see Figure 2C). In this way, we enforced a 2-sec interval between consecutive discrete movements. The TDB trials were 60 seconds long in order to allow for the generation of a similar number of movements in the two block types.

In addition we tested whether the effect of rhythmicity on the transfer of motor learning is similar between adults and children. Two groups of children performed the same task, only these experiments consisted of two blocks, 15 trials each. The two conditions tested were the discrete-small movement (DS) and rhythmic-small movement (RS). One group of children performed the DS condition first, and the other performed the RS condition first.

**Figure 3:**
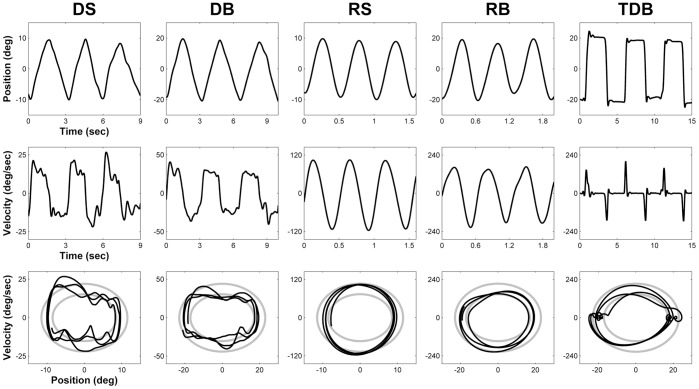
Examples of movement trajectories and phase-plane display for the five experimental conditions. Top panels: position as a function of time. Middle panels: velocity as a function of time. Bottom panel: the display of the allowed range as seen on the computer screen is shown in gray, –and the phase-plane trajectory is shown in black.

Examples of position and velocity traces as a function of time and a phase-plane display for the 5 experimental conditions are shown in Figure 3.

### Data Analysis

Position was recorded as the angular displacement about the elbow joint and filtered using a zero-phase, first-order Butterworth filter with a cutoff frequency of 20 Hz. Trend was removed from the position data, so as to reduce the effects of drift. This was achieved by removing the best straight-line fit from the angular position data. Data were analyzed using MATLAB® (The MathWorks, Natick, MA).

### Movement Error

In order to determine the start and end points of each point-to-point movement in the TDB blocks, we detected the time points in which the velocity trace reached an extreme. For each extreme point we detected the first time before and after that point in which the velocity value was smaller than 5% of the peak velocity. These points were considered as the start and end points of the movement, respectively.

The error in each trial was calculated as the portion of the movement time spent outside the allowed region.

### Harmonicity

**Figure 4:**
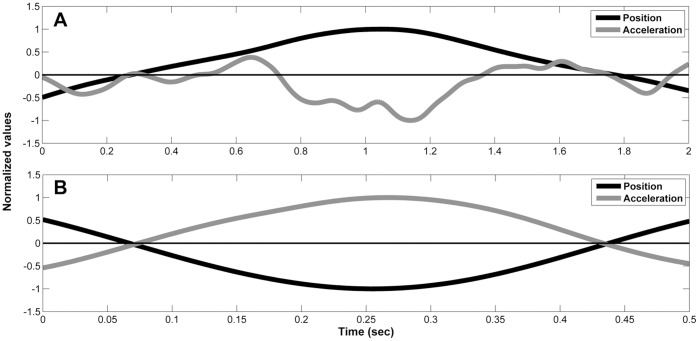
The computation of harmonicity (H). The computation of harmonicity (H) is portrayed for a discrete movement, for which H = 0, in (A) and for a rhythmic movement, for which H = 1, in (B). In both plots, the black line corresponds to position trace of a single half-cycle of movement around a reversal and the gray line corresponds to the acceleration trace.

In order to determine whether the movement is rhythmic or discrete in experiments 1–8, we used the index of harmonicity, a measure based on inflection points in the acceleration trace (see Figure 4). For each half cycle of the movement, between two zero-crossings in the position trace, the index of harmonicity was calculated as follows: in case there was only one peak in the acceleration trace in that half cycle, harmonicity value was set to be one, indicating a rhythmic movement. In case that two or more peaks in the acceleration trace occurred in the half-cycle, harmonicity value was computed as the ratio of the minimum to the maximum absolute values of the acceleration within that half cycle. If within the half cycle the acceleration trace changed sign, harmonicity value was set to zero, indicating a discrete movement [7], [8], [12], [14]. The harmonicity value of a trial was computed as the mean of the individual harmonicity values of each half cycle belongs to that trial. It has been demonstrated that the harmonicity index is a robust indicator of movement type [7].

The convention is that movements with H values lower than 0.5 are considered discrete in nature and the movements with H values higher than 0.5 are rhythmic [3], [4], [7], [8], [15], [16].

### Statistical Analysis

Unless otherwise noted, paired t-test analysis was performed to compare the differences in error in each of the experiments.

The Holm-Bonferroni correction was applied where necessary, to account for multiple comparisons and avoid type-I error.

P values smaller than 0.05 were considered to indicate a significant difference.

## Results

### Harmonicity

For the two discrete blocks in experiments 1–8 (DS and DB), the mean values of H were 0.086±0.01 and 0.095±0.01 (mean±SE), respectively (significantly lower than 0.5, p<0.001) and for the two rhythmic blocks (RS and RB), the mean values of H were 0.71±0.02 and 0.76±0.02 (mean±SE) respectively, (significantly higher than 0.5, p<0.01), confirming that the discrete blocks were indeed discrete and the rhythmic ones rhythmic.

### Effect of Rhythmicity: No Transfer of Learning

**Figure 5:**
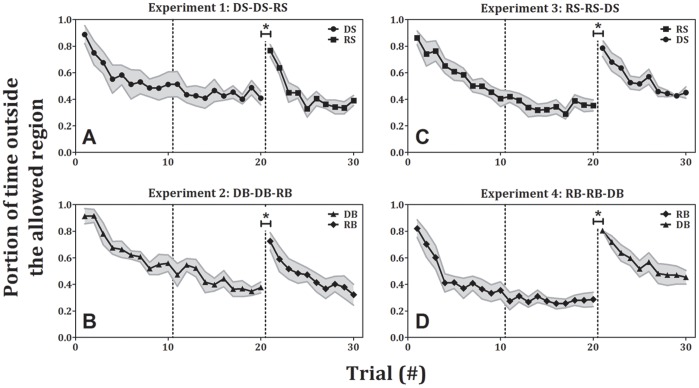
Error as a function of trial number for experiments 1–4 (± standard error). Each shape represents a different condition of the task: circles – discrete small, triangles – discrete big, rectangles – rhythmic small, diamonds – rhythmic big. In all transitions, from discrete movements to rhythmic ones (panels A and B) and from rhythmic movements to discrete ones (panels C and D), there was a significant increase in error.

In experiments 1–4 (Figure 5), participants performed 2 blocks for a total of 20 trials of a certain condition and then 10 more trials with the same amplitude but the other type of movement (e.g., participants who performed 20 trials of small discrete movements (DS) performed in the following 10 trials small rhythmic movements (RS)). All the participants had learned the task in the first two blocks, as the error in the last trial of the second block (0.41±0.05, 0.38±0.04, 0.35±0.04 and 0.29±0.05, mean±SE for DS, DB, RS and RB, respectively) was significantly lower than the error in the first trial of the first block (0.89±0.07, 0.91±0.06, 0.86±0.05 and 0.82±0.07, mean±SE for DS, DB, RS and RB, respectively; p<0.001 for all conditions). The effect of the change in rhythmicity (the transition from the second to the third block, from discrete to rhythmic or vice versa) is evidenced by the significant increase in error from the last trial of the second block to the first trial of the third block in each of these experiments (0.77±0.05, 0.72±0.07, 0.78±0.06 and 0.8±0.01, mean±SE for RS, RB, DS and DB, respectively; p<0.005, for all conditions). Then, once again, the participants had learned the task and significantly reduced their error between the first and the last trials of the third block (0.39±0.04, 0.32±0.08, 0.45±0.04 and 0.45±0.05, mean±SE for RS, RB, DS and DB blocks, respectively; p<0.01 for all conditions). This suggests that there is no transfer of learning across the two movement types. To further explore this, we also examined the effect of practice in one movement type on the performance in the other. We tested whether performing the movements in blocks 1 and 2 reduced the error in block 3, compared to the error in that condition without any prior practice. To that end, for each condition (DS, DB, RS and RB), we compared between the error in the first trial, when the condition appeared in the third block to the error in the first trial when the same condition appeared in the first block (for example, we compared the error in the first DS trial in experiment 3 to the error in the first DS trial in experiment 1). For all the conditions there was no significant decrease in error when they appear in the third block, compared to when they appear in the first block. Since practice on one type of movement (rhythmic/discrete) did not improve performance on the other, we conclude that indeed there was no transfer of learning between the two types of movement.

**Figure 6:**
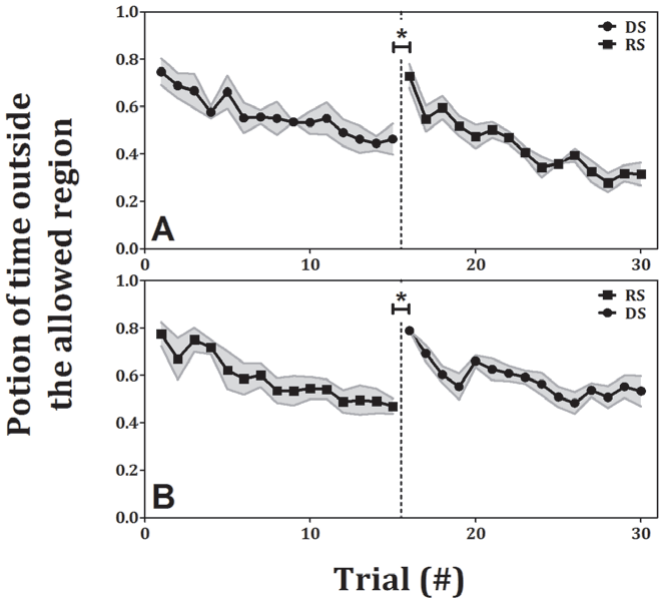
Error as a function of trial number for the children control experiment (± standard error). Each shape represents a different condition of the task: circles – discrete small, rectangles – rhythmic small. In both transitions, from discrete movements to rhythmic ones (panel A) and from rhythmic movements to discrete ones (panel B), there was a significant increase in error.

In the children’s experiment we observe similar behavior. In both conditions, participants had learned the task as the error in the last trial of the first block (0.46±0.07 and 0.47±0.03, mean±SE for DS and RS, respectively) was significant lower than the error in the first trial of that block (0.75±0.06 and 0.77±0.05, mean±SE for DS and RS, p<0.001, p<0.01, respectively). In the transition between blocks, which differed in the type of movement, the error significantly increased again (0.73±0.05 and 0.79±0.01, mean±SE for RS and DS respectively, p<0.001). In addition, there is no decrease in error when they appear in the second block, compared to when they appear in the first block. These finding indicate that there was no transfer of learning between discrete and rhythmic movements, and vice versa, see Figure 6).

### Effect of Amplitude: Asymmetric Transfer of Learning

**Figure 7:**
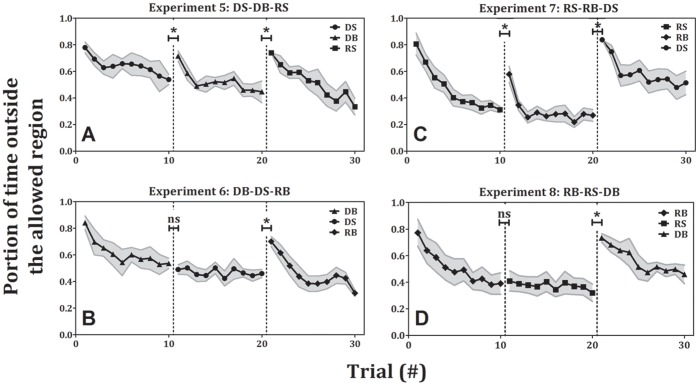
Error as a function of trial number for experiments 5–8 (±standard error). Each shape represents a different condition of the task: circles – discrete small, triangles – discrete big, rectangles – rhythmic small, diamonds – rhythmic big. In the transition from small movements to big movements (panels A and C, first transition), there is a significant increase in error. In the transition from big movements to small movements (panels B and D, first transition), there is no significant change in error. In the transition from discrete movements to rhythmic ones (panels A and B, second transition) and from rhythmic movements to discrete ones (panels C and D, second transition), there is a significant increase in error.

Here we compare the transition between the first block to the second block of experiments 5–8 (Figure 7).

### Small to Big

Panels A and C in Figure 7 demonstrate the transition from small-amplitude movements (DS and RS, respectively) to big amplitude movements (DB and RB, respectively), within the same type of movement (discrete and rhythmic, respectively). The error in the first trial of the second block (0.71±0.04 and 0.59±0.06, mean±SE for DB and RB, respectively) was significantly higher than the error in the last trial of the first block (0.54±0.04 and 0.31±0.02, mean±SE for DS and RS, p<0.01 and p<0.002, respectively). After this increase in error, there was a new learning process and the error in the last trial of the second block (0.45±0.08 and 0.27±0.04, mean±SE for DB and RB, respectively) was significantly lower than the error in the first trial of this block (p<0.05 and p<0.005, respectively). In addition, there was no significant difference in the error when conditions DB and RB were performed in the first blocks (experiments 2 and 4, respectively) compared to when they were performed in the second blocks (experiments 1 and 3, respectively; the comparison was made between the first trials of the first and the second blocks). In other words, practicing on a smaller scale movement did not improve the performance when performing larger scale movement.

### Big to Small

When the transition was from large-amplitude movements (DB and RB) to small amplitude movements (DS and RS, respectively, see Figure 7B and 7D), it appears that the participants considered the second block in these experiments as a continuation of the first block, as evidenced by their reaching a plateau in their ability to improve: (1) the error in the first trial of the second block (0.49±0.04 and 0.41±0.08, mean±SE for block DS and RS, respectively) was not significantly higher than the error in the last trial of the first block (0.54±0.04 and 0.39±0.08, mean±SE for block DB and RB, respectively), and (2) the error differences between the first and the last trials in blocks DS (0.03±0.03 mean±se, Figure 7B) and RS (0.09±0.06 mean±se, Figure 7D; both are the second blocks in the respective experiments) were not significantly different.

### Interaction between the Effects of Amplitude and Rhythmicity

The results from experiments 1–4 demonstrated that when the rhythmicity was changed, without changing the amplitude, there was no transfer of learning (Figure 5). The results from the first transition in experiments 5–8 demonstrated that when the amplitude was changed, without changing the rhythmicity, there was a transfer of learning from large-amplitude movements to small-amplitude movements (Figure 7). In the second transition of experiments 5–8 (from the second to the third block) we changed both the amplitude of the movement and its rhythmicity simultaneously. This change did not allow for a transfer of learning between the blocks, as indicated by a significant increase in the error from the last trial of the second block (0.45±0.08, 0.46±0.03, 0.27±0.04 and 0.32±0.06, mean±SE for experiments 5–8, respectively) to the first trial of the third block (0.74±0.01, 0.7±0.04, 0.84±0.01 and 0.73±0.03, mean±SE for experiments 5–8, respectively, p<0.02, see Figure 7). This was the case even in experiments 5 and 7 (panels A and C in Figure 7) in which the transition was from big movements (discrete and rhythmic, respectively) to small movements (rhythmic and discrete, respectively). Furthermore, in this case too, we found that there was no significant difference for all four conditions (DS, DB, RS and RB) in experiments 5–8 in the first trial between the experiments in which the condition appeared in the third block and the experiments in which the condition appeared in the first block.

### Control Experiment - Truly Discrete Movement

**Figure 8:**
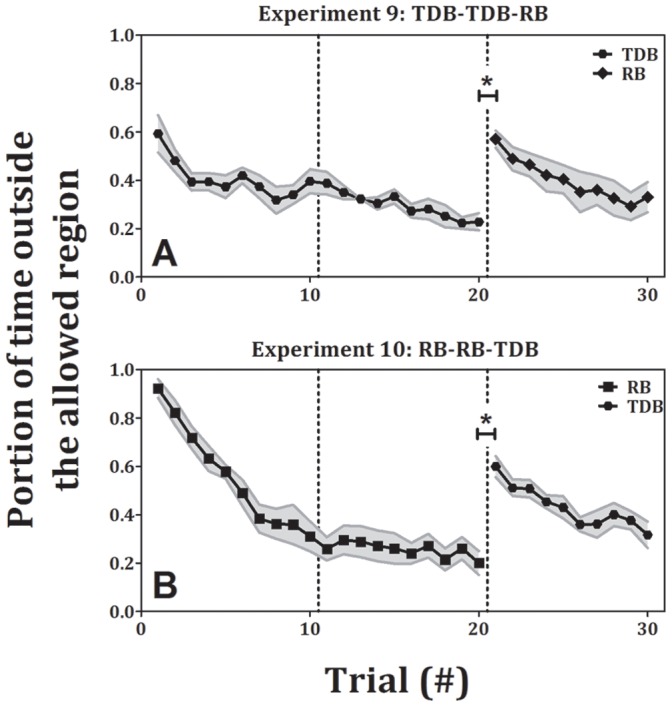
Error as a function of trial number for experiments 9 and 10. (± standard error). Each shape represents a different condition of the task: hexagons – truly discrete big, diamonds – rhythmic big. In the transition from truly discrete movements to rhythmic ones (A) and from rhythmic movements to discrete ones (B), there is a significant increase in error.

In experiments 9 and 10 participants performed two consecutive blocks of TDB or RB movements (10 trials in each block, for a total of 20 trials), followed by 10 RB or TDB trials, respectively. In these experiments, the required amplitude and speed were the same across blocks, and the only difference was the rhythmicity of the movement: In the discrete condition participants had to stop for 2 seconds at each end-point (maximum flexion and maximum extension) and in the rhythmic condition the participants were instructed not to stop until the end of the trial. We verified that there was no significant difference between the TDB and RB movements in terms of the movement amplitude or peak speed. The performance in the control experiments (Figure 8) was similar to the performance in experiments 1–4. Again, all the participants had learned the task in the first two blocks, as the error in the last trial of the second block (0.23±0.04 and 0.2±0.05, mean±SE for TDB and RB, respectively) was significantly lower than the error in the first trial of the first block (0.59±0.08 and 0.92±0.04, mean±se, p<0.01 and p<0.001 for TDB and RB, respectively). When a change in rhythmicity occurred (the transition from the second to the third block), a significant increase in error between the first trial of the third block (0.57±0.04 and 0.6±0.04, mean±SE for RB and TDB, respectively) and the last trial in the second block in the two experiments was shown (p<0.001). Then, once again, the participants learned the task and significantly reduced their error between the first and the last trials of the third block (0.33±0.06 and 0.32±0.05, mean±SE for RB and TDB, respectively, p<0.05). In the control experiments there is no significant difference between the first TDB trial when it appeared in the third block to the first TDB trial that appeared in the first block. When comparing the error in the first RB trial that followed 20 TDB trials (experiment 9) to the error in the first RB trial when RB was the first block (experiments 4, 8 and 10), we found mixed results. A one-way ANOVA test revealed a significant difference among the first RB trials in the four experiments (p<0.01). However, post-hoc analysis showed that there were no significant differences in error between the first RB trial that came after TDB practice (0.57) and the first RB trial in two out of the three blocks in which RB appeared first (0.92±0.03, 0.82±0.03 and 0.77±0.03, mean±se, p<0.01, p = 0.12 and p = 0.6 for experiments 10, 4 and 8, respectively).

## Discussion

We tested the effects of movement rhythmicity and amplitude on the ability to transfer motor learning in a spatiotemporally defined task. We found no transfer of learning from rhythmic to discrete movements. This result was not affected by a change in the peak speed of the movement, as we found that there was no transfer of learning from rhythmic to discrete movement regardless of the how discrete movements were defined (point-to-point movements with distinct inter-movement pauses or low-frequency, non-harmonic movements). We also found there was no transfer from discrete to rhythmic movements, when the discrete movements were low frequency, non-harmonic movements, but a partial transfer when these movements were point-to-point ones. Additionally, we found that there was an asymmetric transfer of learning from large-amplitude movements to small-amplitude movements within the same type of movement (rhythmic/discrete).

### Rhythmic vs. Discrete Movements

We tested whether there is a transfer of learning from rhythmic to discrete and from discrete to rhythmic movements. Using a learning-transfer paradigm, we can examine the extent of overlap between two tasks and infer whether the mechanism generating these two types of movements are at least partially separate. The relation between discrete and rhythmic movements has been a topic of interest in the last few decades. Studies have sought to establish mathematical models for the generation of discrete and rhythmic models [17], [18], [19], [20], explore the relationship between these movements behaviorally [1], [2], [3], [4], and map the neural processes involved in the generation and the control of these two types of movements and the relationship between them [5].

The motor control literature offers three possible models for the control of rhythmic and discrete movements. The first model considers rhythmic movement as the basic movement and discrete movement as merely a truncated rhythmic movement [21], [22]. The second model suggests that rhythmic movement is a concatenation of discrete movements, which constitute the fundamental movement type [3]. These two models imply that there is one mechanism that generates and controls the two types of movement, with other circuits possibly responsible for starting and stopping the rhythmic generator or repeating the use of the discrete actions. The third model suggests two separate (or partially separate) control mechanisms, one for the control of rhythmic movements and one for the control of discrete movements [1], [2], [5].

We showed even though there was an increase in error in the transition from a truly discrete movement to highly rhythmic one, the error on the big rhythmic movement was, in 1 out of 3 cases, significantly lower after the practice on the truly discrete movement, compared with no practice. Ikegami et al. [2] results show a partial transfer between discrete and rhythmic movements when the dwell time between two discrete movements was two seconds (as we used in this study). These two findings suggest at least partial transfer from discrete to rhythmic movements. It is worth conducting a similar experiment but with a longer dwell time between two consecutive movements in order to explore whether this partial transfer still exists. Howard et al. [1] showed that in a force-field perturbation task, when different force fields were associated with each type of movement (rhythmic/discrete), participants could adapt to both force perturbations without interference between them. Their results suggest that when the type of movement acted as a contextual cue, there was no interference in the parallel learning processes of the two force fields. In our study, it is possible that the rhythmicity served as a different context; therefore, even though we found transfer of learning from large to small movements within the same movement type (rhythmic/discrete), we found no such transfer when the movement type changed concurrently with the amplitude.

Imaging studies explored which brain areas are involved in the generation of rhythmic and discrete movements. Schaal et al. [5] used functional magnetic resonance imaging (fMRI) imaging and showed that rhythmic movement activated a small number of unilateral primary motor areas (such as the primary motor cortex(M1), the supplementary motor area (SMA) etc.) whereas discrete movement activated additional contralateral nonprimary motor areas and a very strong bilateral activity in cerebellum. In the Spencer et al. [23] study, patients with cerebellar lesions exhibited no deficits in the production of continuous rhythmic movement, but had difficulty in matching the target intervals in the discontinuous movements. Their results suggest an important role for the cerebellum in timing of discrete movements. Future studies should implement this study paradigm in the context of brain imaging in order to further explore the specific areas required for the generation of the two types of movements.

An alternative interpretation of the results may be that the frequency of the different conditions played a role in facilitating or hindering transfer of learning. In the main experiment the distinction between discrete and rhythmic movements was based on the frequency of the movement, while in the control experiment, both movements had the same frequency. We have shown [12] that frequency is the controlled variable when performing rhythmic movements. The frequency control hypothesis can explain why there was decrease in error in the first RB trial after practicing with 20 TDB trials comparing to the first RB trial (without any practice before). In the context of motor learning as flexible combination of primitives that encode the kinematics of the limb (e.g., [24]), our results suggest that different frequencies are encoded by different primitives. There is a possibility that the subjects learned the frequency of the movement in the truly discrete trial and when they switch to the rhythmic movement they could use that information in order to better succeed in the task. When the two movements differ in frequency (discrete vs. rhythmic), subjects could not use the learned knowledge about the frequency of the movement in order to improve their performance in the other task.

### Big vs. Small Movement Amplitude

Braden et al. [25] found a transfer in sequence learning between large-scale movements to smaller-scale movements. In another study Dean et al. [11], found asymmetric transfer of learning between movements with large space between the targets to movements with smaller space between the targets. Our results agree with the results of these studies, as we too found an asymmetric transfer of learning from large scale movements to small scale movements within the same type of movement (rhythmic/discrete). For a given movement frequency, big amplitude movements require higher velocity, acceleration, and greater force production. Due to these kinematic and dynamic factors, when performing larger movements in comparison to small-scale movements, more resources may have to be recruited in order to succeed in the task. Brown and Cooke [26] found that when performing flexion-extension movements about the elbow during a step-tracking task, the duration of the initial agonist burst was increased with the increase in movement amplitude. They suggested that the CNS has two mechanisms for the generation of large-scale movements: increasing the amount of activity of the initial agonist burst and generating a second pulse.

### Conclusions

We found that (1) there was no transfer of learning from rhythmic to discrete movements and a partial transfer of learning from discrete to rhythmic movements, which suggests that the two movements types do indeed draw on at least partially separate neural resources, as previously demonstrated using imaging technology [5]; these results add to the imaging findings in demonstrating that the existing partial overlap in the involved brain structures is not sufficient to allow a complete transfer between the two movement types; (2) Within each movement type (rhythmic/discrete) there was transfer of learning from movements with large amplitude to movements with smaller amplitude and not vice versa. This asymmetry may result from the higher mechanical and neuronal demands that are needed for the generation of large scale movements.

The ability to transfer a skill is important for both theoretical research and for the design of protocols for rehabilitation of disorders and injuries that affect the motor system, such as stroke and traumatic brain injury. Studying patterns of learning can give us insights regarding how different aspects of the movement (e.g., rhythmicity, amplitude) are stored and represented in the motor memory. Furthermore, in a rehabilitation protocol for Parkinson Disease patients for example, extensive practice bigger than usual movements may later be transferred to smaller, regular movements in order to achieve faster more accurate movements (see [27]).
